# C2-3 Fracture Dislocation and Bilateral Vertebral Artery Occlusion Without Neurological Injury: A Case Report

**DOI:** 10.7759/cureus.5538

**Published:** 2019-08-30

**Authors:** John W Kiessling, Eric Whitney, Brian Fiani, Yasir R Khan, Deependra Mahato

**Affiliations:** 1 Neurosurgery, Desert Regional Medical Center, Palm Springs, USA

**Keywords:** vertebral artery occlusion, cervical spine subluxation, cervical spine trauma

## Abstract

A 27-year-old female involved a motor vehicle collision as the restrained driver presented to the ER with agonal breathing and a Glasgow Coma Scale (GCS) of 3. Radiographic imaging demonstrated C2-3 craniocaudal dislocation, bilateral C2 comminuted pedicle fractures extending through the transverse foramina, complete bilateral vertebral artery occlusion, and negative signs of stroke with MRI. After halo immobilization, surgical stabilization, and medical treatment the patient was discharged and at her six-month follow up she was without neurological deficit.

## Introduction

Cervical spine fracture-dislocation continues to be a devastating injury that typically results in significant spinal cord injury (SCI) in a large portion of affected patients [1-2]. Subluxation appears to occur typically between the C4 and C7 segments with some studies showing only 24% occurring in segments above C4 [1-4]. Specifically, dislocation at the C2-3 level is considered a rare level for subluxation to occur [5]. In addition, vertebral artery injury (VAI) in the setting of cervical spine trauma is an appreciated entity and while the incidence is reported at 0.5%, this is believed to be an under-diagnosed injury as a majority are asymptomatic [6-8]. Here, we report a case where the patient suffered both significant C2-3 craniocaudal fracture dislocation with complete bilateral vertebral artery occlusion but suffered no significant neurological injury.

## Case presentation

**Figure 1:**
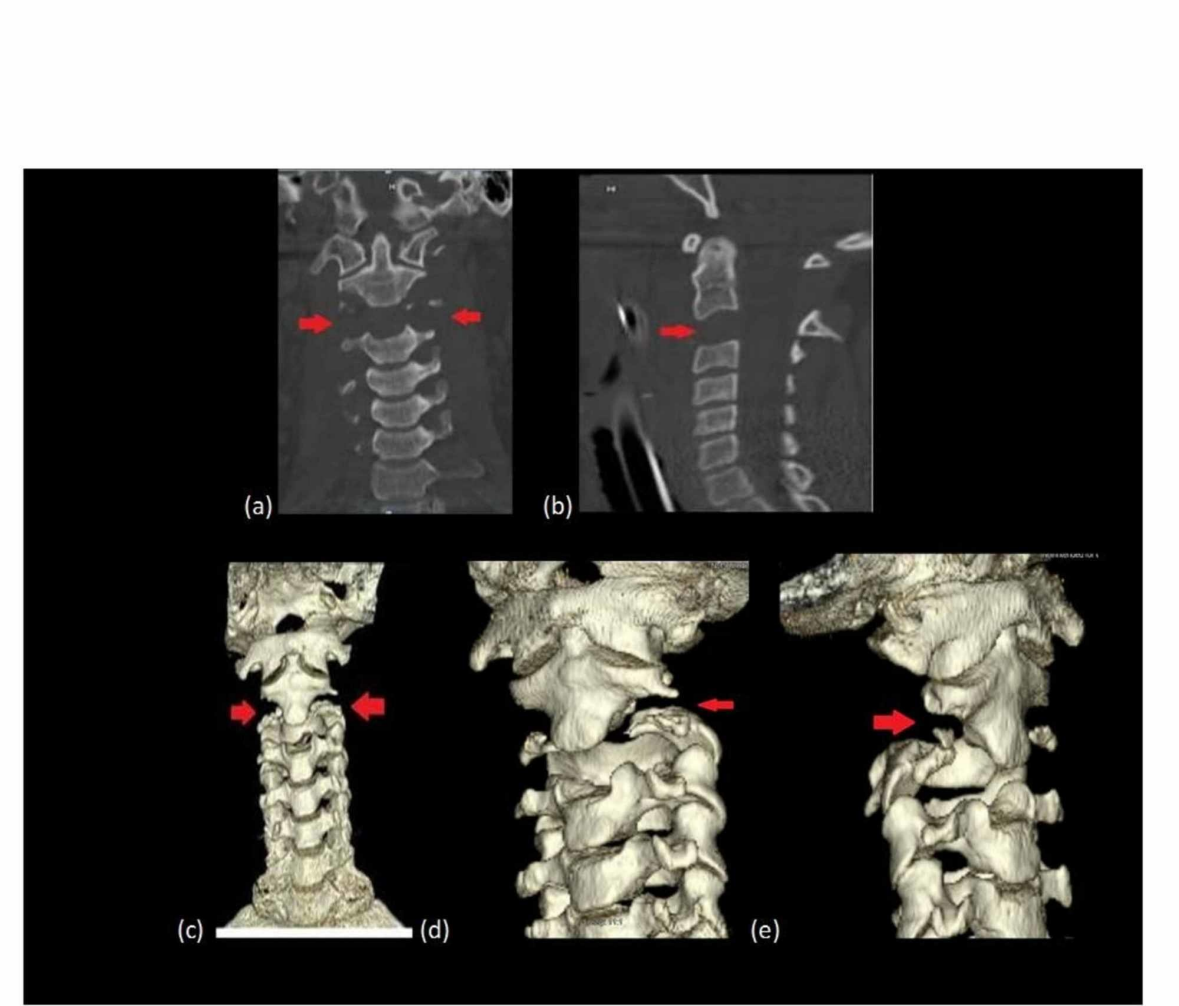
CT of the cervical spine: (a) coronal, (b) sagittal, (c) CT 3D reconstruction (d) and (e) 3D oblique view. All views with red arrows show cranial caudal dislocation of C2 on C3.

**Figure 2:**
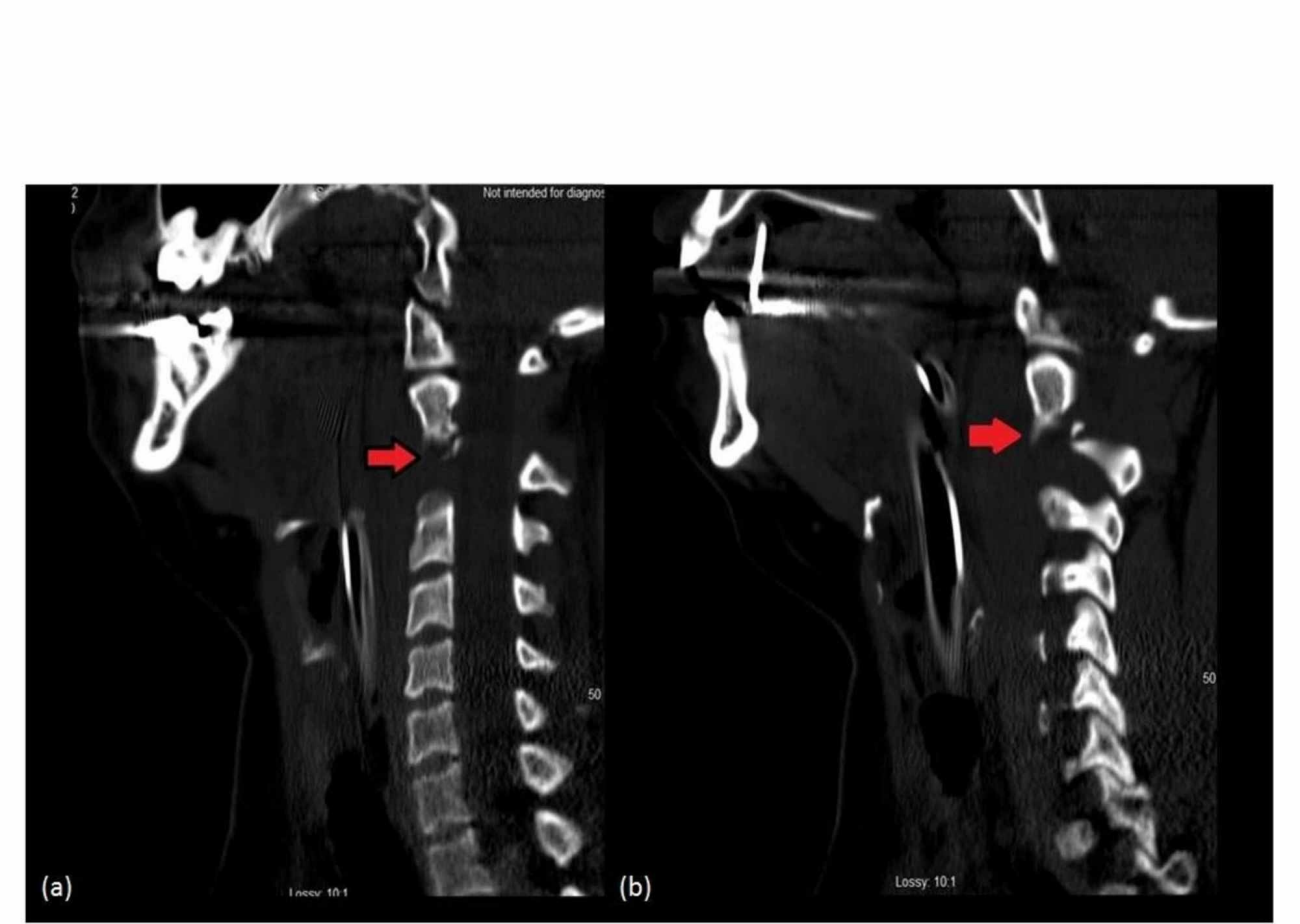
Sagittal view (a) left and (b) right : CT cervical spine with a red arrow showing C2 pedicle fracture.

**Figure 3:**
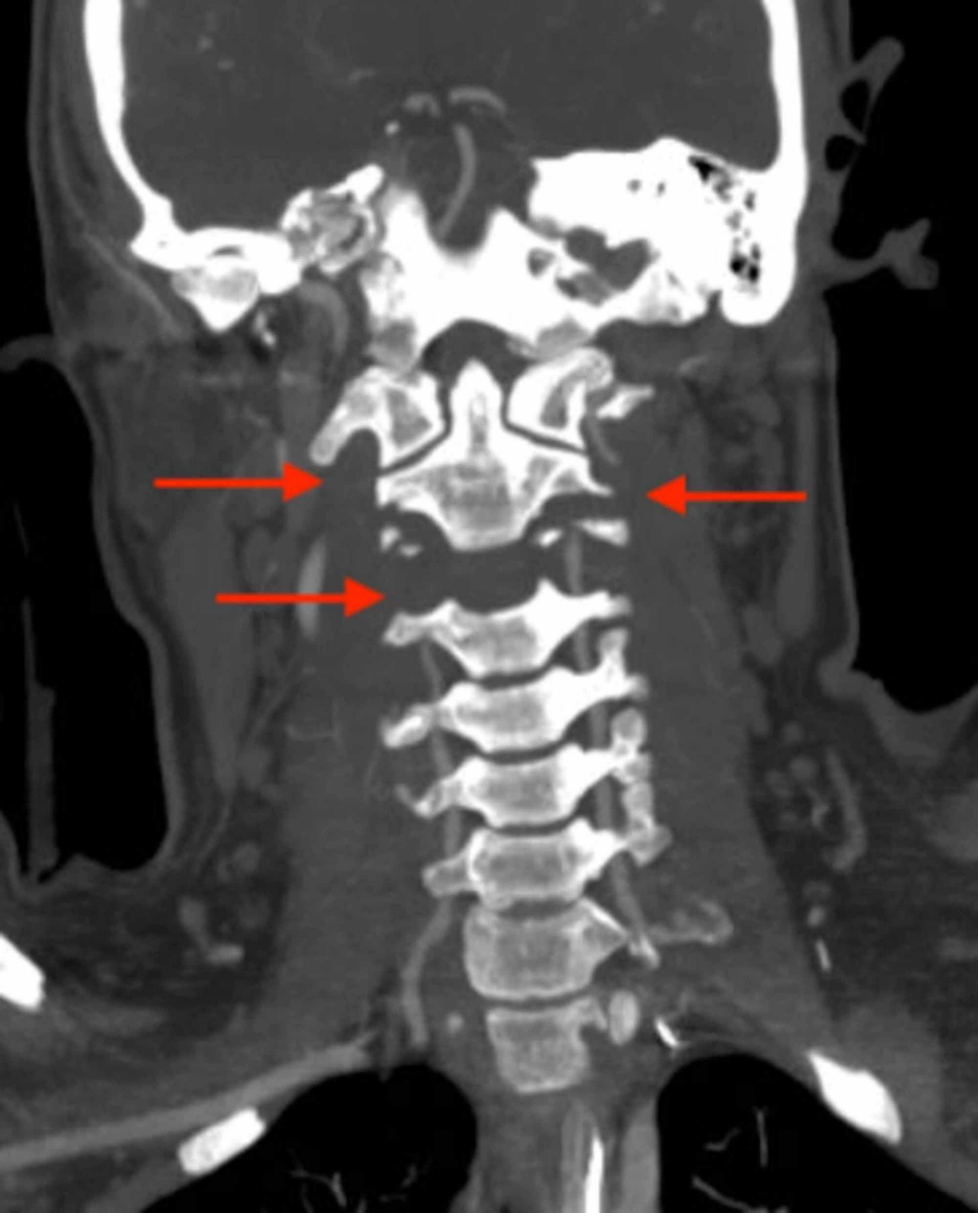
CT angiography of the cervical spine: coronal view red arrows show bilateral vertebral artery injury at the level of C2.

**Figure 4:**
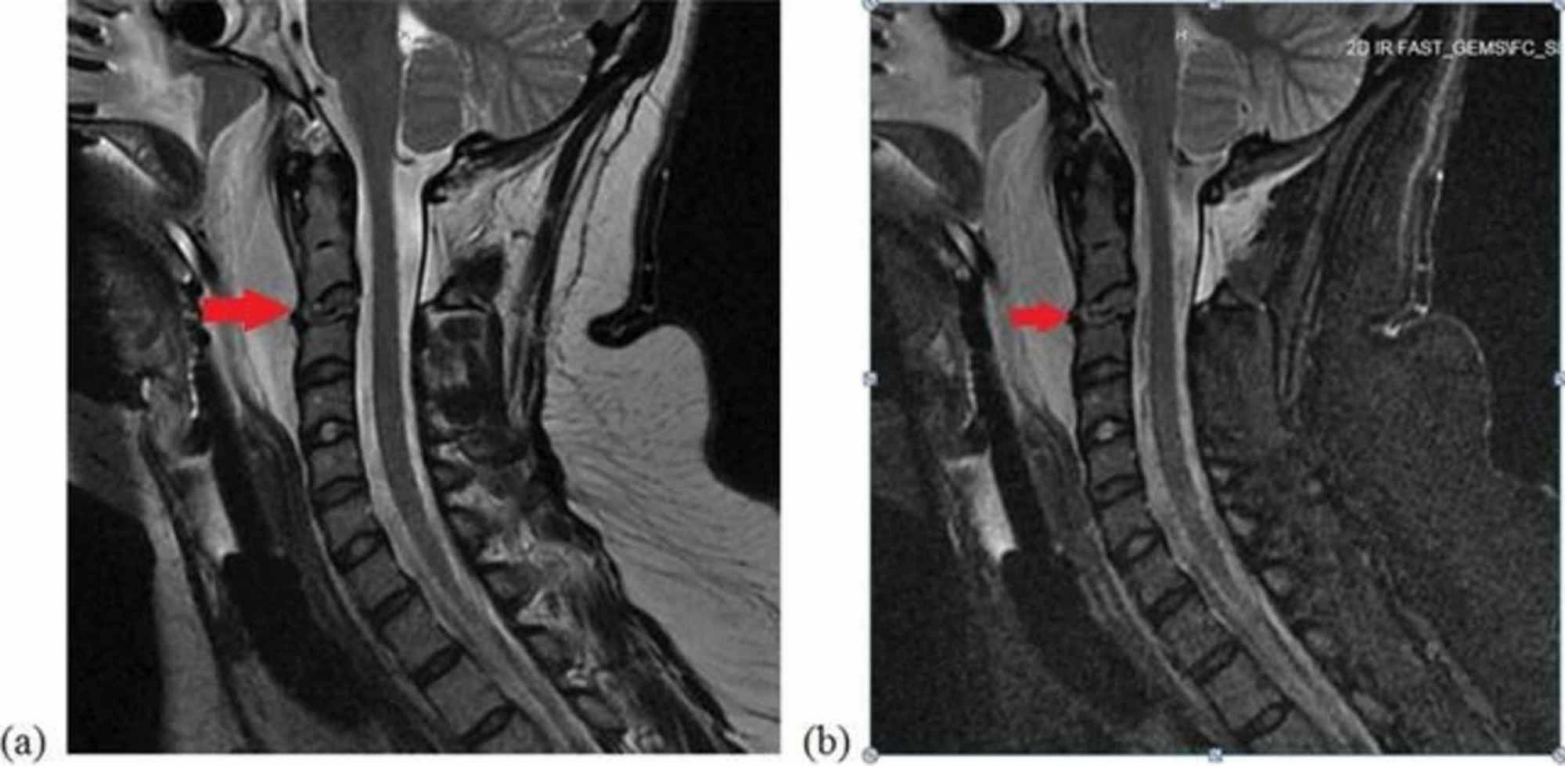
MRI of the cervical spine. (a) T2-Weighted sagittal view and (b) STIR sagittal view of the cervical spine with red arrows show C2-3 intervertebral disc injury.

A 27-year-old female was brought into the trauma bay after being involved in a motor vehicle accident as the T-boned restrained driver. In the field she was reported to have had a prolonged extrication from the vehicle as well as agonal gasping and a low Glasgow Coma Scale (GCS) of 3. On arrival in the trauma bay, the patient was remained at GCS 3 as per initial assessment and was rapidly intubated using rapid sequence intubation. After initial trauma evaluation, the patient was hemodynamically stable and taken for imaging. CT scan of the cervical spine (Figures 1-2) showed marked craniocaudal separation of C2-3 by 8 mm, bilateral C2 comminuted pedicle fractures extending through their respective transverse foramina, and significant prevertebral swelling. CT angiography of the head and neck revealed bilateral VAI at the level of C2 with complete nonopacification of the right vertebral artery to the craniocervical junction and the left vertebral artery to the C1 level (Figure 3). MRI of the cervical spine showed C2-3 intervertebral disc injury but no apparent disruption of the either the anterior longitudinal ligament (ALL) or posterior longitudinal ligament (PLL) (Figure 4). No cord edema or posterior atlantoaxial ligamentous injury was seen but a 2.2 cm prevertebral hematoma from the retropharyngeal space to the C5 level was noted. It was also noted that there had been almost 50% reduction of the C2-3 distraction between the CT and the MRI of the cervical spine respectively. MRI of the brain showed no signs of acute pathology, including cerebellar or brainstem infarction.

Once back in the trauma bay, her paralytics were reversed with sugammadex and the patient’s exam improved to a GCS 11T. It was noted that the patient was having significant horizontal nystagmus but she was able to follow commands to test extraocular movements in all directions and they were grossly intact. She was able to follow commands in the right upper extremity by showing a thumbs-up and she would wiggle her toes to command in the right lower extremity but would not follow other commands for strength testing. However, she had strong localization with mild stimulation in the bilateral upper extremity with at least 4+/5 strength in all muscle groups and withdrew briskly and purposefully in the bilateral lower extremities. No clear, consistent lateralizing deficit was detected. She was hyper-reflexic throughout the bilateral upper and lower extremities to bicep, tricep, brachioradialis, patellar, and achilles deep tendon reflexes and she had bilateral Hoffman’s reflexes and clonus. Her rectal tone was intact with positive bulbocavernosus reflex, indicating no signs of spinal shock. Attempts to verify sensation were unreliable given the patient’s agitation off sedation.

 Given the significant reduction in seen distraction despite rigid collar immobilization, the patient was placed in a halo vest immobilizer in order to prevent further movement. At the time, no clear SCI was evident but she would not follow commands in all extremities. With her intermittent ability to follow commands, her upper motor neuron signs, and her significant cervical instability, the possibility of SCI was high and there was concern for SCIWORA. Thus, her mean arterial pressure (MAP) was kept greater than 85 mmHg as per our institution’s SCI protocol. Her other injuries were only significant for a right clavicular fracture and she had no solid or hollow organ injury.

**Figure 5:**
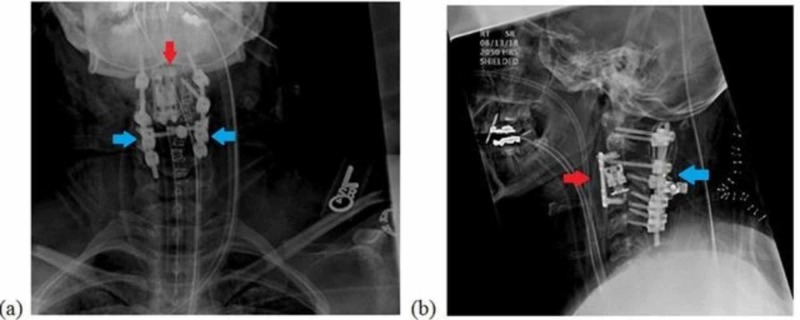
Postoperative cervical radiographs: (a) AP view and (b) lateral view with red arrows show a 12 mm vertebral body replacement cage was placed and a 26 mm anterior plate and blue arrows showing posterior instrumentation of C1-C4 with sublaminar wiring between C1 and C2.

 Given that the patient’s bilateral VAI showed no-flow through the involved segments, MRI brain was obtained. This showed no signs of stroke, and widely patent retrograde flow through the basilar artery below the bilateral posterior inferior cerebellar arteries (PICA). With this information, the neurointerventional team recommended no further neuro-endovascular imaging, intervention, or antiplatelet therapy. Once it was determined that the patient did not appear to be having any evolving or active strokes and did not require emergent neuro-endovascular intervention, she was taken to the operating room for C1-C4 posterior instrumentation and posterolateral arthrodesis, C1-2 sublaminar wiring with posterior fusion, as well as C3-C4 laminectomy. Intraoperatively, distraction was noted between C1-2 and C2-3 and the patient was kept in the halo throughout the entire surgery. No changes in intraoperative neuromonitoring (will delete: deficits), for either somatosensory evoked potential (SSEP) or motor evoked potential (MEP), were noted either before, during, or after surgery. Once postoperative fluoroscopic images showed good placement of instrumentation and interval stabilization of the C2-3 distraction, the halo orthosis was completely removed. The following day, the patient was taken for a C2-3 anterior cervical discectomy and fusion (ACDF) through a right-sided approach. Intraoperatively, frank complete disruption of the ALL and PLL was found with destruction of the C2-3 intervertebral disc. A large prevertebral hematoma was encountered and evacuated as well as a hematoma within the C2-3 disc space itself but no active bleeding was encountered. Once the disc was removed, a 12 mm vertebral body replacement cage was placed and a 26 mm anterior plate was secured over it. Ideal placement was confirmed both with intraoperative fluoroscopy and postoperative cervical radiographs and the patient was kept in a rigid cervical collar (Figure 5). Once again, no intraoperative neuromonitoring changes (SSEP and MEP) were detected.

Postoperatively, the patient’s exam remained the same with brisk 4+/5 localization in the bilateral upper and lower extremities but poor effort when undergoing voluntary strength and sensation testing. Once extubated, the patient was noted to be hoarse and laryngoscopy found left vocal cord paralysis. She underwent a steroid taper but was unable to pass multiple swallow evaluations and had a percutaneous gastrostomy (PEG) tube placed. However, after extubation and the weaning of multiple medications, the patient began following commands reliably and demonstrated full strength in the bilateral upper and lower extremities with intact sensation. At the time of her six-month follow up, the patient has had her PEG tube removed, oral diet advanced, continued to demonstrate no neurological deficit, and her follow up imaging has displayed intact and stable instrumentation. She did not undergo any following vascular imaging as she has remained asymptomatic from her vascular injuries and is a young women of child-bearing age who is continuing to have children.

## Discussion

Cervical spine injury has the potential to be a very morbid or lethal injury. In particular, upper cervical injury is considered the most likely to result in lethal injury. However, the levels of injury typically considered are at the craniocervical junction or at C1-2 [4]. These types of upper cervical injuries are more likely to be seen in the pediatric population due to the ligamentous laxity still present at a young age [4, 9]. Reports of specifically C2-3 dislocation injuries are sparse and some consider this due to the likelihood of sudden death resulting from the associated SCI [4]. To our knowledge, there has only been one other case reported in a 57-year-old-male of a C2-3 dislocation injury that had mild neurological injury [5]. In striking similarity to our case, the patient also had bilateral VAI but was unable to obtain an MRI of the cervical spine or brain secondary to a noncompatible cardiac pacemaker, thus presence or absence of post-VAI strokes is unknown.

 Our patient had little in the way of contributing polytrauma, thus we were able to obtain good exams with minimal confounding injuries despite her inability to follow commands reliably. While initially on exam she appeared to have signs of cerebellar or even brainstem involvement, these rapidly reversed and no strokes were seen on MRI. Throughout her course, her response to mild noxious stimuli was always indicative of a lack of significant SCI, which was corroborated with her MRI showing no cord signal changes whatsoever. It should be noted that as compared to other cervical distraction injuries which are typically considered on involvement of facet dislocation, our patient did not have that component. With bilateral C2 pedicle fractures, the distraction occurred primarily at the C2-3 disc where it was found to be completely destroyed intraoperatively. It is evident on imaging that there is distraction between the posterior arches of C1-2 but the atlantoaxial membrane remained intact. However, despite the MRI findings, complete disruption of the ALL and PLL was found intraoperatively. When considering why the patient was largely unaffected by her injury, the lack of facet involvement may be a reason. In Quarrington et al., where 226 patients with cervical subluxation or dislocation were retrospectively reviewed, they reported on the suggestion that “concomitant fracture of the posterior elements at the level of dislocation may reduce the risk of SCI by increasing the space available for spinal cord” [1, 10-11]. However, in their own results, they did not see that concomitant fracture or spinal canal diameter was predictive of SCI. In contrast to the reason posited for the minimal SCI in Machinis et al., our patient was young and had no previous cervical laminectomies at the involved levels that may have possibly conferred protection via previous canal decompression [5]. With regards to the morphology and mechanism of her distraction injury, we believe that despite being struck on the side where a more horizontal or translational injury would be expected, she somehow experienced a more purely craniocaudal tensile force that lacked any significant horizontal, anterior-posterior, or translational component.

**Figure 6:**
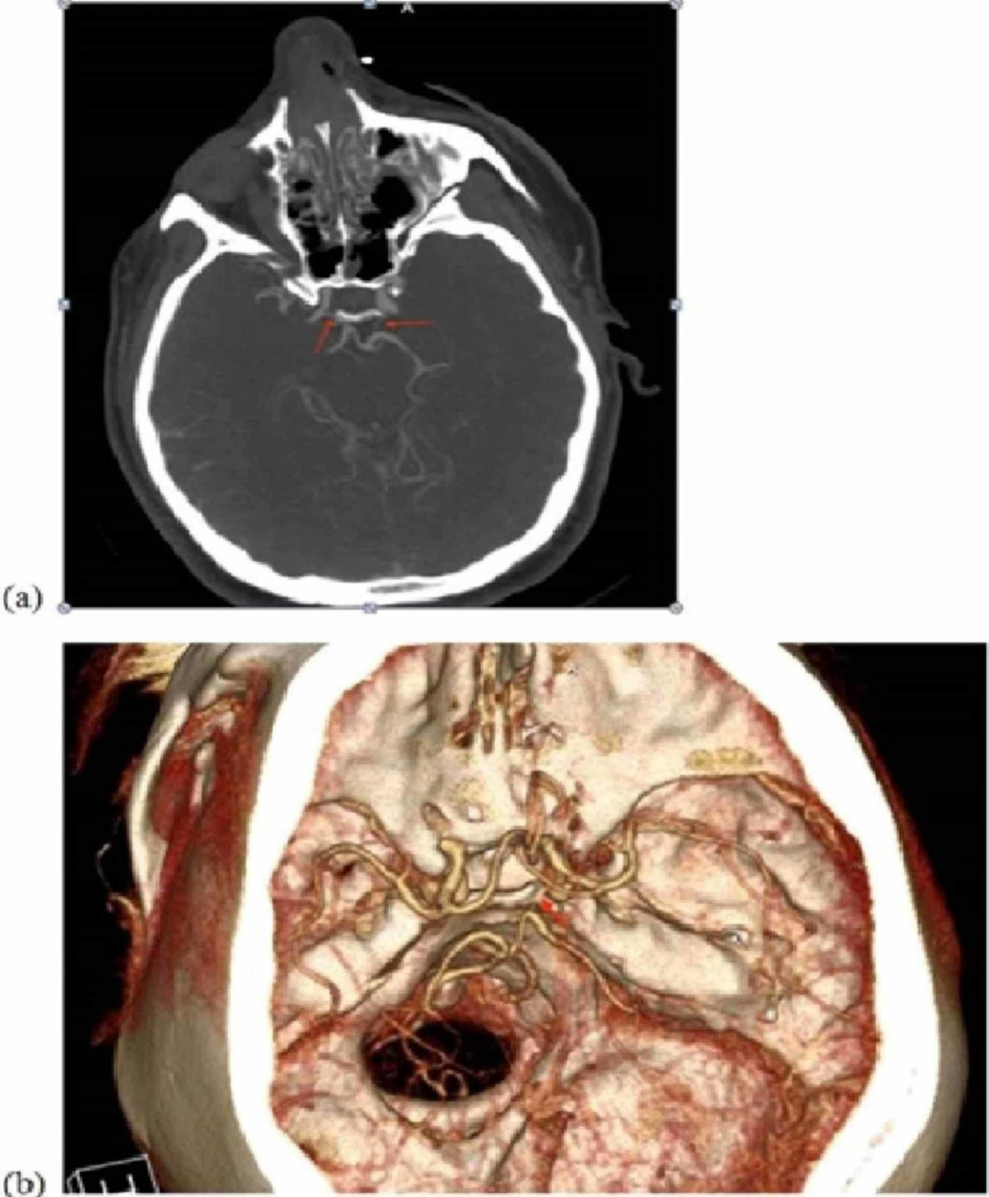
CT angiography of the brain: (a) axial cut with arrows indicating the right and left posterior communicating arteries, respectively. (b) CT angiography reconstruction demonstrating a patent right posterior cerebral artery.

 Perhaps most importantly, our patient also suffered bilateral vertebral artery occlusions. While she initially presented with nystagmus that was concerning for impending severe neurological injury, this time involving the cerebellum or brainstem rather than the spinal cord, she recovered extremely quickly and her MRI brain was without any acute pathological findings. She did have left vocal cord paralysis after extubation but it is unclear if this is due to her injury or her intubation. Our approach for her ACDF was from the right and would not have likely impacted her left vocal cord. Patients presenting with bilateral VAI with no concurrent symptoms are extremely rare and in the case of Machinis et al., they posited the presence of fetal circulation [5]. Our patient did neither undergo any diagnostic cerebral angiography nor undergo any endovascular intervention for bilateral VAI and this was due to the rapid resolution of her nystagmus, the presence of a negative MRI of the brain, and continued integrity of her physical exam. On CT angiography, it does not appear the vertebral arteries are transected, despite the finding of a large prevertebral hematoma. By the grading scale put forward by Biffl et al. for blunt cerebrovascular injury, our patient had a Grade IV injury as there was bilateral occlusion but no transection with free extravasation [12]. In their report, a grade IV VAI had a stroke incidence of 28% [12]. However, for all those found, they were all unilateral injuries [12]. There are very few reports on the presence of bilateral vertebral artery cervical trauma but some authors posited that bilateral VAI is uncommon as the injury mechanism leading to VAI is typically from an asymmetrical force [13]. Others have concluded that bilateral VAI likely carries a 100% mortality, though they do note that the data is very limited [14-15]. In our case, the patient had a patent right posterior communicating artery and a present, though much less robust, left posterior communicating artery (Figure 6). There was excellent retrograde reperfusion of the basilar artery all the way past the bilateral PICA, likely the reason for the lack of stroke or continued symptoms. However, without angiography, the presence or absence of persistent fetal circulation remains unknown.

 The surgical treatment of upper and subaxial cervical spine injury remains a topic of debate and the most recent guidelines from the American Association of Neurological Surgeons and the Congress of Neurological Surgeons recommend either anterior or posterior fixation and fusion, with neither being superior. They both conclude that regardless of the approach taken, adequate decompression of the spinal cord should be ensured [15-16]. In our particular case, we placed the patient in a halo orthosis once it was seen that the C2-3 segment was hypermobile despite a rigid cervical collar and strict cervical spine precautions, as demonstrated by the changes in distraction length. The goal was to prevent motion as much as possible while operative intervention was pending. Surgically, given the hypermobility at the injured level, we performed posterior instrumentation first with the patient in the halo. Posthalo placement radiograph demonstrated adequate interval stabilization and alignment at C2-3 and with posterior instrumentation performed first, we were more comfortable with an anterior fusion without being concerned for new anteroposterior translation or changes in the level of distraction. Furthermore, it allowed us to performed decompressive laminectomies at C3-4 in preparation for the planned anterior surgery so as to allow the maximal amount of canal space should any movement or instrumentation failure occurs. A significant amount of disc, ALL, and PLL injury was found anteriorly and with the degree of distraction, a vertebral body replacement that is usually reserved for corpectomies had to be used to stabilize the anterior and middle cervical columns. At the patient’s most recent six-month follow up, she has been doing very well with no neurological symptoms and has been graduated out of her rigid cervical collar.

## Conclusions

High cervical spinal trauma, particularly dislocations and subluxations, remains an injury highly associated with significant morbidity if not lethality. Here we report a unique case involving a significant craniocaudal C2-3 fracture dislocation with extensive disc, anterior, and middle column ligamentous injury, however, with minimal neurological symptoms. In addition, she suffered bilateral acute vertebral artery occlusions yet remains asymptomatic with no signs of stroke or cerebellar or brainstem injury. While the lack of distinct facet involvement may have contributed to the lack of neurological injury, our patient still presented with surprisingly minimal neurological symptoms despite the severity of her bony, ligamentous, and vascular injury.
